# Adiposity Mediates the Association between the Dietary Inflammatory Index and Markers of Type 2 Diabetes Risk in Middle-Aged Black South African Women

**DOI:** 10.3390/nu11061246

**Published:** 2019-05-31

**Authors:** Asanda Mtintsilana, Lisa K. Micklesfield, Elin Chorell, Tommy Olsson, Nitin Shivappa, James R Hebert, Andre P. Kengne, Julia H. Goedecke

**Affiliations:** 1 South African Medical Research Council/University of the Witwatersrand Developmental Pathways for Health Research Unit, Department of Paediatrics, Faculty of Health Sciences, University of the Witwatersrand, Johannesburg 2193, South Africa; mtintsilana.asanda@gmail.com (A.M.); Lisa.Micklesfield@wits.ac.za (L.K.M.); 2 Division of Exercise Science and Sports Medicine, Department of Human Biology, University of Cape Town, Cape Town 7725, South Africa; 3 Department of Public Health and Clinical Medicine, Umeå University, SE-901 87 Umeå, Sweden; elin.chorell@umu.se (E.C.); Tommy.G.Olsson@umu.se (T.O.); 4 Cancer Prevention and Control Program, University of South Carolina, 915 Greene Street, Suite 200, Columbia, SC 29208, USA; shivappa@mailbox.sc.edu (N.S.); jhebert@sc.edu (J.R.H.); 5 Department of Epidemiology and Biostatistics, University of South Carolina, 915 Greene Street, Suite 400, Columbia, SC 29208, USA; 6 Connecting Health Innovations, LLC, 1417 Gregg Street, Columbia, SC 29201, USA; 7 Non-Communicable Diseases Research Unit, South African Medical Research Council, Francie Van Zijl, Parow Valley, Cape Town 7505, South Africa; Andre.Kengne@mrc.ac.za

**Keywords:** DII, diet-induced inflammation, obesity, VAT, mediation, T2D risk, South African women

## Abstract

The dietary inflammatory index (DII^®^), a validated tool used to measure the inflammatory potential of the diet, has been associated with metabolic disorders in various settings, but not in African populations. The aim of this study was to investigate whether the DII is associated with markers of type 2 diabetes (T2D) risk, and if this association is mediated by adiposity and/or low-grade inflammation, in black South Africa women. Energy-adjusted-DII (E-DII) scores were calculated in 190 women (median age, 53 years) from the Birth-to-Twenty plus cohort using a validated food frequency questionnaire. Fasting glucose, insulin, HbA1c, and inflammatory cytokines were measured, and an oral glucose tolerance test performed. Basic anthropometry and dual-energy x-ray absorptiometry-derived body fat, including estimate of visceral adipose tissue (VAT) area, were measured. E-DII scores were associated with all markers of T2D risk, namely, fasting glucose and insulin, HbA1c, HOMA2-IR, two-hour glucose and Matsuda index (all *p* < 0.05). After adjusting for age, measures of adiposity, but not inflammatory cytokines, mediated the association between E-DII and markers of T2D risk (*p* < 0.05). Measures of central obesity had proportionally higher (range: 23.5–100%) mediation effects than total obesity (range: 10–60%). The E-DII is associated with T2D risk through obesity, in particular central obesity, among black middle-aged South African women.

## 1. Introduction

The rapid increase in the prevalence of non-communicable diseases (NCDs), such as type 2 diabetes (T2D), has become a major health concern, particularly in developing countries, such as South Africa (SA) [1,2,3]. South Africa had a diabetes prevalence of 5.5% in 2017 [3]. In 2016, diabetes was the second leading cause of mortality in SA after tuberculosis, accounting for 5.5% of all deaths in both males and females [2]. When classified by sex, diabetes-attributed mortality was worse in females (7.2%) than in males (4.0%), and ranked first and fifth as the cause of death in females and males in 2016, respectively [2].

The prevalence of obesity and insulin resistance (IR), major risk factors for T2D, is higher in black SA women than in white SA women [4,5,6]. Of great concern is that urban black SA women are at even greater risk of NCDs compared to their rural counterparts [7,8]. Urbanization, which is often accompanied by unhealthy diets and low levels of physical activity, has been identified as the main driver of obesity in developing countries [8,9,10,11]. Urban black SA women who follow a typical Western diet characterized by a high intake of fat, sugar and processed meat, and low intake of fruit and vegetables, are more susceptible to NCDs than rural black women who still follow a traditional diet, which is high in fiber, fruit, vegetables and low in animal products [8,10,11].

The relationship between unhealthy diets and NCDs can be attributed to numerous factors, including diet-induced inflammation [12,13]. Indeed, several studies suggest that unhealthy diets may be involved in the pathogenesis of NCDs through their effects on increasing low-grade inflammation [12,13,14], while healthier diets, such as the Mediterranean diet, which are high in fruits, vegetables and fish, are associated with lower levels of inflammation [12,13,15]. Low-grade inflammation is characterized by high circulating levels of pro-inflammatory cytokines, such as c-reactive protein (CRP), tumor necrosis factor- alpha (TNF-α), interleukins (e.g., IL-6), and infiltration of macrophages in insulin-dependent tissues, which may lead to IR and, subsequently, to T2D [16,17].

The dietary inflammatory index (DII^®^), is a literature-derived dietary tool that measures the inflammatory potential of an individual’s diet [14]. Participants with higher DII (i.e., positive values) scores are categorized as having a more pro-inflammatory diet and those with lower DII (i.e., negative values) score represent a more anti-inflammatory diet [14]. The DII has been validated with several inflammatory markers, such as CRP and interleukins [18]; and has recently been associated with markers of glycaemia, namely, fasting glucose, HbA1c, post-load glucose and IR in non-African populations [19,20,21].

Moreover, participants with higher DII scores were at greater risk of developing prediabetes and T2D compared to participants with lower DII scores [20,21]. Notably, the association between DII and markers of T2D has not been studied in a black African population, whose lifestyle (i.e., diet and socioeconomic status (SES)) differs from white and Hispanic populations. SES (both low and high) and sociocultural factors have thus, through their effects on unhealthy diets and eating behaviors, been associated with NCD risk in black SA communities [22,23]. Furthermore, the black SA population still experiences inadequate micronutrient intake [10,11], in particular micronutrients with putative anti-inflammatory properties, such as vitamin A, niacin, iron and zinc. In contrast, energy intake has not undergone any major change [10,11], suggesting that the quality of the diet, rather than the quantity of food consumed, and changes in energy expenditure may explain the high obesity levels, and, in turn, the susceptibility to T2D risk in urban black SA women.

Use of the DII may contribute to our understanding of the role of diet-induced inflammation in the pathophysiology of T2D, while linking it to markers of low-grade inflammation and obesity. Indeed, the DII has been associated with markers of low-grade inflammation and measures of adiposity (i.e., BMI) and these risk factors have also been suggested to mediate the association between DII and markers of T2D in non-African populations [19].

The aim of this study was to determine whether dietary-induced inflammation, measured by the DII, is associated with markers of T2D risk, and if this association is mediated by adiposity and/or inflammatory markers, in middle-aged and older black SA women.

## 2. Materials and Methods

### 2.1. Study Design

This cross-sectional study was conducted between 2015 and 2016 and included 190 black female caregivers of the original Birth to Twenty plus (BT20+) cohort, living in Soweto, Johannesburg [24]. The female caregivers were invited to participate if they fulfilled the following inclusion criteria: (1) Female caregiver of the original cohort; (2) provided stored serum samples and blood analyte data (criteria for the larger study); (3) less than 65 years of age; (4) HIV negative on testing; (5) not currently pregnant or lactating. Of those tested (*n* = 221), nine women were excluded from the statistical analyses, due to unreliable energy intake data (i.e., excessive energy intake (≥30,000 kJ), *n* = 4), lack of food frequency questionnaire (FFQ) data (*n* = 2) and lack of blood samples (*n* = 3). Furthermore, 22 diabetic participants (based on self-report and/or use of T2D medication) were excluded from the analyses, resulting in a final sample size of 190 participants.

The study was approved by the Human Research Ethics Committee (Medical) of the University of the Witwatersrand (M010556 and M150530). The procedures and risks associated with the study were explained to the participants and they all signed the consent form prior to participation in the study. All testing procedures were performed at the South African Medical Research Council (MRC)/University of the Witwatersrand (WITS) Developmental Pathways for Health Research Unit (DPHRU) at the Chris Hani Baragwanath Hospital in Soweto Johannesburg.

### 2.2. Body Composition

Weight and height were measured in subjects wearing only lightweight clothing and without shoes, using a standard scale and stadiometer. Waist (level of umbilicus) and hip (largest gluteal area) circumferences were measured in triplicate and the average was used for statistical analyses.

The International Diabetes Federation waist circumference (WC) threshold (women: 80 cm) was used to calculate abdominal obesity (expressed as a percentage) [25]. BMI was calculated as weight (kg)/height (m)^2^ and classified according to WHO criteria; underweight (<18.5 kg/m^2^), normal weight (18.5–24.9 kg/m^2^), overweight (25–29.9 kg/m^2^), or obese (≥30 kg/m^2^) [26]. Whole body composition was measured using dual energy x-ray absorptiometry (DXA; Hologic Discovery-W (S/N 71201), Bedford, MA, USA; software version 13.4.2:7), and included subtotal (whole body minus head) fat mass (expressed in kg and as a percentage of sub-total fat mass, %FM). Abdominal visceral adipose tissue (VAT) was estimated using algorithms included in the DXA, and has been shown to correlate with clinical computed tomography [27]. Body composition of participants exceeding the scan field limits was calculated using the arm-replacement [28]. The coefficient of variations were 1% and <2% for fat-free soft tissue mass and total fat mass, respectively.

### 2.3. Socio-Demographic Assessment

A demographic questionnaire, written in English, was administered in the participant’s language of choice (i.e., English, IsiXhosa, IsiZulu, SeSotho, Sepedi, SeTswana, TshiVenda, XiTsonga, SiSwati) and included measures of SES, including housing density, asset index, household food insecurity, marital status, educational assessment, and employment. Housing density was defined as the number of persons per room living in the household. Asset index was based on 12 items (e.g., motor vehicle, washing machine, TV) reflecting individual and household wealth. Food insecurity was assessed using a validated nine question Household Food Insecurity Access Scale [29]. The responses to the nine-questionnaire scale are summed to create a food security score, with a maximum score of 27. The higher the score the more food insecurity the household experienced, whereas the lower the score the less food insecurity experienced in the past four weeks [29]. Education was categorized by grades passed. Participants were categorized as being married or not, employed or unemployed. Moreover, questions on lifestyle factors, including smoking and alcohol intake were recorded. Physical activity and sedentary behavior were measured using ActiGraph GT3X-Plus triaxial accelerometers (ActiGraph GTX3+, ActiGraph LLC, Pensacola, Florida) and activPAL devices (activPAL3c, PAL Technologies Ltd., Glasgow, UK), respectively, as described previously [30].

### 2.4. The Dietary Inflammatory Index (DII^®^)

**Figure 1:**
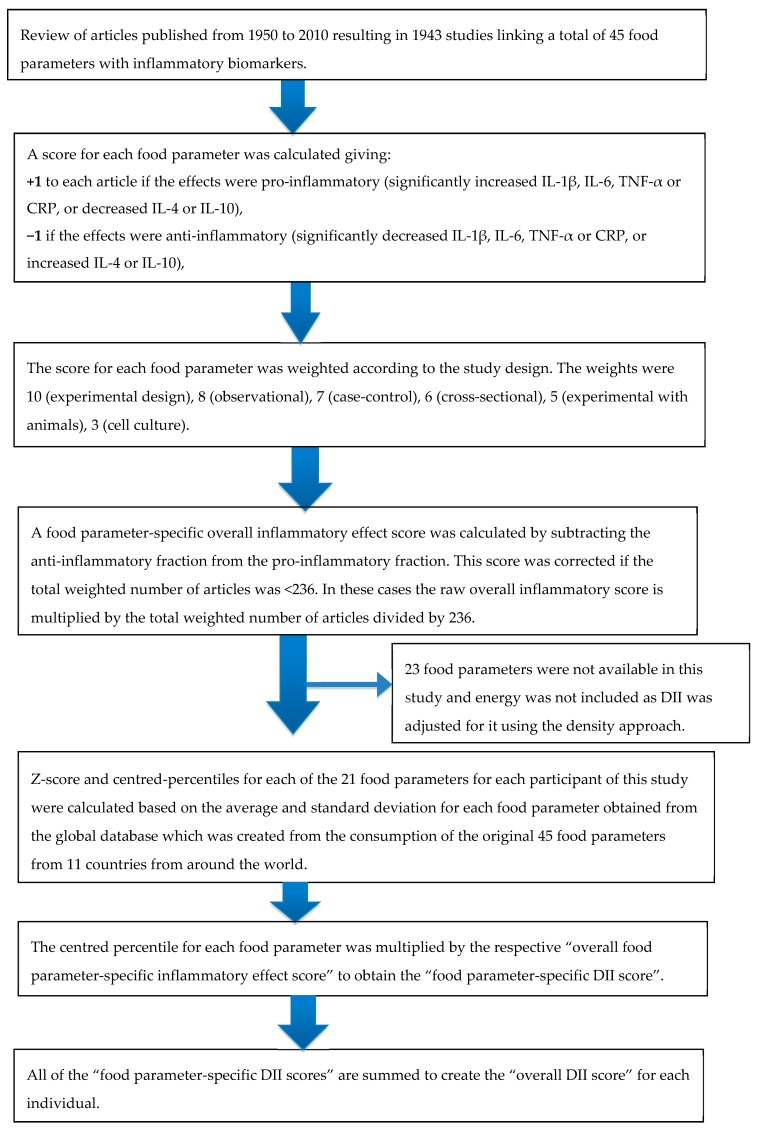
Sequence of steps in creating the energy-adjusted- DII (dietary inflammatory index) in middle-aged black South African women.

The DII scores for all the participants in the current study were calculated from the dietary intake data collected using a seven-day food frequency questionnaire [31,32]. The development and validation of the DII have been described in detail elsewhere [14,18]. The DII is a literature-derived, validated tool used to measure the inflammatory potential of the diet based on extensive literature search performed to identify peer-reviewed primary research articles published through 2010 that reported the association between various dietary factors and six inflammatory markers (IL-1β, IL-4, IL-6, IL-10, TNFα, and CRP). A total of 1943 qualifying articles were reviewed, indexed, and scored to derive the component-specific inflammatory effect score for 45 dietary factors (i.e., components of DII), comprising macronutrients and micronutrients, as well as some bioactive components [14]. These FFQ-derived food and nutrient intake data were first adjusted for total energy (i.e., per 1000 kilocalories) and standardized by creating a z score for each component using mean and standard deviation data from a global energy-adjusted dietary database comprised of dietary intake from 11 populations living in different regions of the world. To reduce the effect of right-skewing the z scores for each DII component were converted to proportions (i.e., values from 0 to 1) and then each proportion was centered by doubling and subtracting 1. From this, the energy-adjusted standardized dietary intake (expressed as centered proportions) was then multiplied by the corresponding literature-derived inflammatory effect score for each DII component. Individual scores from each DII component were then summed to determine the overall E-DII score for each individual [14]. A higher (i.e., more positive) score indicates a more pro-inflammatory diet and a lower score (i.e., more negative) represents a more anti-inflammatory diet. The DII scores can range between 7.98 (maximally pro-inflammatory) and −8.87 (maximally anti-inflammatory) [14]. Steps involved in the DII calculations are described in Figure 1.

For the current study, a total of 21 out of 45 food parameters were available in our dietary intake database, and were thus used for the analysis of the DII score. The 21 food parameters included protein, fat, SFA, monounsaturated fatty acid (MUFA), polyunsaturated fatty acid (PUFA), carbohydrate, cholesterol, alcohol, fiber, iron, magnesium, Zinc, vitamin A (retinol equivalent), thiamine, riboflavin, niacin, vitamin B6, vitamin B12, vitamin C, vitamin D and vitamin E. Participants with E-DII scores greater than 0 were classified as the positive E-DII group (reflecting pro-inflammatory diet; *n* = 83) and those with E-DII scores less than 0 were classified as negative E-DII group (indicating anti-inflammatory diet; *n* = 107). There were no participants with E-DII score equal to 0.

### 2.5. Biochemical Analysis

Blood samples were drawn in the fasted state (12 hours, overnight) for the subsequent determination of glycated hemoglobin (HbA1c), plasma glucose and serum insulin concentrations. Thereafter, all participants completed a standard oral glucose tolerance test (OGTT). In brief, participants ingested 75g of anhydrous glucose dissolved in 250 mL water, within 5 minutes. Following the glucose ingestion, blood samples were taken at 30, 60, 90 and 120 min. The samples were centrifuged at 3000 rpm for 10 min at 4 °C, the plasma was stored at −20 °C for subsequent analysis of glucose concentrations, and the serum was stored at −80 °C for the analysis of insulin concentrations and inflammatory markers. Plasma glucose concentrations were analyzed on the Randox RX Daytona Chemistry Analyzer using enzymatic methods (Randox Laboratories Ltd., London, UK). Serum insulin assays were analyzed on the Immulite^®^ 1000 Immunoassay System (Siemens Chemiluminescent Healthcare GmbH, Henkestr, Germany). HbA1c levels were measured on whole blood samples using the D-10™ Hemoglobin Analyzer (Bio-Rad Laboratories, Inc. Hercules, CA, USA). Inflammatory cytokines, namely, IL-6, IL-1ra, IL-8, IL-10, IL15, monocyte chemotactic protein (MCP)1, interferon (IFN) gamma, and TNF-α were measured using Milliplex MAP MAG Human Cytokine kit (Merck, Johannesburg, South Africa) and xMAP technology (Luminex, Austin, Texas) according to the manufacturer’s instructions. Out of the eight cytokines measured, only TNF-α, 1L-8 and MCP-1 were detectable in all the samples, while the rest were below the detectable range, and therefore not included in the analyses.

Insulin resistance (IR) was calculated from fasting glucose and insulin using the Homeostasis Model Assessment (HOMA2-IR) calculator v2.2.3 [33]. Fasting and OGTT glucose and insulin data (0, 30, 60, 90 and 120) were used to calculate insulin sensitivity using the Matsuda Index web calculator [34]. Participants with missing glucose and insulin data from the OGTT (*n* = 17) were excluded from this parameter.

### 2.6. Statistical Analysis

**Figure 2:**
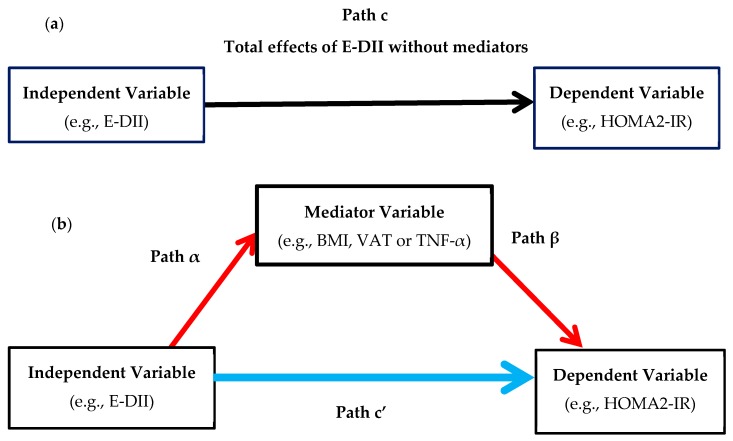
Represent a single mediator model used to test the association between the energy-adjusted-dietary inflammatory index (E-DII) (i.e., independent variable) and markers of type 2 diabetes (T2D) (i.e., dependent variable), body composition and inflammatory cytokines as mediators: (**a**) Path c, represents the simple total effect of E-DII on markers of T2D, without adjusting for mediators; (**b**) Represents the direct (Path c’) and indirect effects (product of Paths α and β, αβ) of E-DII on markers of T2D after controlling for the effect of the mediator. BMI, body mass index; E-DII, energy-adjusted-dietary inflammatory index; HOMA2-IR, Homeostasis Model Assessment- insulin resistance; TNF-α, tumor necrosis factor alpha; VAT, visceral adipose tissue.

The Shapiro-Wilk test was used to assess the distribution of continuous variables. Normally distributed data are presented as mean ± standard deviation (SD), skewed variables are represented as medians and 25th–75th percentiles, and categorical data are presented as frequency (%). Moreover, Levene’s robust test was used to test for equal variance between groups. Differences in measures of SES, lifestyle/behavioral factors, body composition and markers of T2D risk between the positive and negative E-DII groups were examined using the Student’s t test and the Wilcoxon rank-sum (Mann-Whitney), or the Chi square test or Fisher exact test as appropriate. To determine whether the E-DII was associated with markers of T2D risk (fasting glucose and insulin, HbA1c, HOMA2-IR, two-hour glucose and OGTT-derived insulin sensitivity), or whether the association was mediated by adiposity (WC, BMI, VAT and body fat mass) and/or inflammatory markers (TNF-α, IL-8 and MCP-1), a simple mediation analysis was completed (Figure 2a,b) [35]. In each model, an independent, a dependent and a mediator variable were included in order to calculate the unstandardized regression coefficients and generate three outputs, namely, total, direct and indirect effects [36]. Path c, represents the simple total effect of E-DII on markers of T2D without adjusting for mediators (Figure 2a). Path α represents the regression coefficient between the independent variable (i.e., E-DII) and the mediator variable (e.g., VAT) (Figure 2a). The regression coefficient of Path β represents the effect of the mediator variable on the markers of T2D (Figure 2b). The product of regression coefficients α and β (αβ) represents the mediated effect (indirect effect) of E-DII on markers of T2D through the mediator variable (Figure 2b). Path c’ represents the direct effect of E-DII on markers of T2D after controlling for the effect of the mediator (Figure 2b).

Bootstrapping (5000 replications) was used to generate normal-based bootstrapped confidence intervals around the indirect effect. Full or complete mediation is present when the total effect and indirect effects are significant, while the direct effect (path c’) is non-significant. When the total and indirect effects are significant, and the direct effect remains significant, partial or incomplete mediation has occurred. Inconsistent mediation is present when neither total nor direct effect is significant and αβ is significant. The proportion of the mediation effect was calculated using the following equation: [αβ/(αβ + c’)]. The Matsuda Index was modelled as an inverse variable in order to calculate the proportion of mediation effect; thus, the regression coefficients are positive instead of negative. All single mediation analysis models were adjusted for age. All data were analyzed using Stata^®^ (Version 13.1, Statcorp, College Station, TX, USA). A test was considered statistically significant if *p* ≤ 0.05.

## 3. Results

### 3.1. Participants’ Characteristics According to E-DII Score

The median (25th–75th percentiles) age of the total sample was 53 (48–59) years. The E-DII ranged from +1.92 (most pro-inflammatory) to −2.51 (most anti-inflammatory). Participants were grouped based on whether they had a positive (*n* = 83; 43.7%) or negative (*n* = 107; 56.3%) E-DII score (Table 1).

While BMI and total body fat did not differ between groups, waist circumference, WHR and VAT were higher in the positive E-DII group compared to the negative E-DII group. Furthermore, participants with a positive E-DII score had higher two-hour glucose and TNF-α levels than those with a negative E-DII score (*p* < 0.05). There were no significant differences in any of the fasting or OGTT-derived measures of T2D risk, and inflammatory markers (IL-8 and MCP-1) did not differ between the groups (Table 1).

Comparisons of measures of SES and lifestyle/behavioral factors, and nutrient intakes between the two E-DII score groups are presented in Tables S1 and S2, respectively. There were no differences in any of the measures of SES and lifestyle/behavioral factors, including physical activity and sedentary behaviors between the two E-DII score groups (Table S1). A median score of 9 out of 12 possible household items (e.g., motor vehicle and washing machine) was reported for the participants. Furthermore, the participants scored an average of 4 (range: 0–9) on the Household Food Insecurity Access Scale, indicating that the participant/household experienced less food insecurity for the measured period.

Energy and carbohydrate intake (except when carbohydrate is expressed as a percentage of energy intake), alcohol and cholesterol levels did not differ between groups (Table S2). In contrast, protein was lower, but total fat, SFA, MUFA and PUFA (both in grams and percentage of energy intake) were higher in the positive E-DII score group than the negative E-DII score group (all *p* < 0.05) (Table S2).

Vitamins A, B12 and D, and riboflavin did not differ between the groups, whereas fiber intake and the rest of the minerals (e.g., iron, magnesium and zinc) and vitamins (e.g., vitamin B6, C and E) were lower in the positive DII score compared to the negative DII score group (*p* < 0.05) (Table S2).

### 3.2. Associations between E-DII and Markers of T2D Risk in Black SA Women

The unstandardized regression coefficients of the total effect of E-DII on markers of T2D risk without (Path c) and with body composition and inflammatory cytokines as mediators (Path c’) are presented in Table 2 and Table 3, respectively. All the models were adjusted for the potential effects of age. E-DII was positively associated with all markers of T2D risk, namely, fasting glucose and insulin, HbA1c, HOMA2-IR, two-hour glucose levels and Matsuda Index (i.e., OGTT-derived insulin sensitivity) (all *p* < 0.05).

The association between the proposed mediators and E-DII (path α) are presented in Table 4, while the associations between the potential mediators and the outcome, markers of T2D risk (path β), are presented in Table 5. Path α showed that E-DII had a significant association with all body composition measures, whereas only TNF-α associated with E-DII among the pro-inflammatory cytokines (Table 4). Analysis of Path β, showed that all the body composition measures were significantly associated with fasting insulin, HOMA2-IR and Matsuda Index Table 5). Furthermore, both WC and VAT (measures of central obesity) were positively associated with HbA1c and two-hour glucose load, with only VAT significantly associated with fasting glucose. In contrast, none of the inflammatory cytokines were significantly associated with markers of T2D risk (Table 5).

### 3.3. Associations between E-DII and Markers of T2D Risk in Black SA Women with Body Composition as Mediators

The direct (Path c’) and indirect effects (product of Paths α and β, αβ) of E-DII on markers of T2D risk, through body composition, including the proportion of mediation by the body composition measures also are presented in Table 2. A consistent finding was that VAT was a significant mediator of the association between E-DII and all markers of T2D risk, including fasting glucose and insulin, HbA1c, HOMA2-IR, two-hour glucose and Matsuda Index (all *p* < 0.05). Both WC and BMI mediated the association between E-DII and fasting insulin, HOMA2-IR and Matsuda Index (all *p* < 0.05). Body fat mass mediated the association between E-DII and fasting insulin and HOMA2-IR (all *p* < 0.05). The proportion of the effect mediated by measures of central obesity (i.e., VAT and WC) was higher (23.5–100%) than the proportion mediated by measures of total obesity, including BMI and body fat mass (10–60%). Notably, VAT mediated most of the association between E-DII and markers of T2D risk, in particular for measures of insulin resistance (HOMA2-IR) and sensitivity (Matsuda Index) (proportion of mediation: 100%). Furthermore, the proportion of the mediation effect for HOMA2-IR and Matsuda Index was higher (40–100%) than for measures of glucose tolerance (e.g., fasting and two-hour glucose load). For instance, the mediating effects on the association between E-DII and HOMA2-IR were: For VAT (100.0%), WC (80.0%), BMI (60.0%) and body fat mass (48.0%) (Table 2).

Direct effects (Path c’) between E-DII and T2D markers were not significant after adjusting for WC, BMI and VAT (Table 2). Similar findings were reported for body fat mass, except for the association between E-DII and two-hour glucose load.

### 3.4. Direct and Indirect effects of E-DII on Markers of T2D in Black SA Women with Inflammatory Cytokines As Mediators

The associations between E-DII and markers of T2D before and after adjustment for the effect of inflammatory cytokines on T2D risk are shown in Table 3. The inflammatory cytokines did not have any significant mediating effect on the association between E-DII and markers of T2D risk (Table 3). After adjusting for the inflammatory markers, E-DII was directly associated with fasting glucose (except for TNF-α) and insulin, HbA1c, HOMA2-IR, two-hour glucose load and Matsuda Index (Table 3) (*p* < 0.05).

## 4. Discussion

This is the first study, to our knowledge, to suggest a link between diet-associated inflammation, as assessed by E-DII, and markers of T2D in a sample of middle-aged black SA women. Notably, we show that this association is mediated by adiposity, in particular central obesity (VAT and WC). Accordingly, measures of central adiposity were higher in participants with a positive E-DII score, reflecting a pro-inflammatory diet, compared to those with a negative E-DII score, which reflected an anti-inflammatory diet. While circulating TNF-α concentrations were higher in the positive E-DII group than the negative E-DII group, circulating inflammatory markers did not mediate the association between E-DII and markers of T2D in middle-aged black SA women.

Typical components of a Western diet, including high intakes of fat and red meat, and low intakes of vitamin A-rich fruit and vegetables, are linked to inflammation and a higher E-DII score [12,13] and have been associated with T2D and CVD risk in urban black SA populations [9,10,11]. These results suggest that pro-inflammatory foods are part of the habitual diet of urban black SA populations, and thus implicated in the pathophysiology of T2D in this population. Although the exact biological mechanisms implicated in the association between diet-induced inflammation and the development of T2D are not fully understood, we propose that excessive intake of pro-inflammatory foods may enhance fat accumulation, increase total and central obesity, in particular VAT, and subsequently induce an inflammatory profile, which is often reported in an obese state [16,17,37]. To substantiate this notion, the direct associations between E-DII and markers of T2D, after adjusting for measures of body composition, in particular VAT, were no longer significant. In contrast, the direct associations between E-DII and markers of T2D remained significant, after adjusting for the inflammatory cytokines. This suggests that a pro-inflammatory diet has a close association with measures of obesity, in particular for pro-inflammatory adipose tissues, such as VAT, and that the presence of obesity is more detrimental than that of low-grade inflammation in the development of T2D risk in this population.

Furthermore, the proposed mechanism for the development of an obese and/or inflammatory state in the presence of a pro-inflammatory diet is supported by the significantly higher measures of central adiposity and TNF-α levels in the positive E-DII score group compared to the negative E-DII group. Accordingly, obesity, in particular central obesity-related inflammation, has been implicated in the development of T2D [16,17,38]. In line with these findings, we reported that VAT mediated most of the association between E-DII and markers of T2D in comparison to other measures of adiposity. The higher proportion of mediation by measures of central obesity, in particular VAT, compared to measures of total adiposity (BMI as a proxy) might be explained by VAT having a higher inflammatory profile and higher rates of lipolysis than other adipose tissue sites [36,39,40]. Further, the anatomical position of VAT relative to the liver, leading to delivery of excess free fatty acids and pro-inflammatory cytokines (e.g., TNF-α) via the hepatic portal system directly to the liver will impair hepatic insulin signaling [36,39,40]. Indeed, ectopic fat accumulation in the liver has been shown to impair many biological functions of the liver, and has been associated with hepatic IR, increased hepatic glucose production and reduced insulin-stimulated hepatic glucose uptake, thus increased risk for T2D [38,39,40].

Our finding that the association between diet-induced inflammation (assessed by the E-DII) and the risk of developing T2D is mediated by adiposity is notable, as only one study, to our knowledge, has reported both adiposity and low-grade inflammation as mediators in the association between diet-induced inflammation and markers of T2D in a European population [19]. However, the two studies are not easily comparable as two different versions of the DII were used; we used a new version of the DII with the updated scoring algorithm. Nonetheless, in our study, low-grade inflammation did not mediate the association between E-DII and markers of T2D. This can be explained by the non-significant association between inflammatory cytokines and markers of T2D risk, thus making a mediation effect “impossible”. Moreover, we used three separate inflammatory cytokines, while a total/summary score of six inflammatory cytokines, including CRP, IL-6, 1L-8 and TNF-α was used as a marker of low-grade inflammation in the previous study [19].

Interestingly, out of the three inflammatory cytokines used in our analyses, only TNF-α was significantly different between the positive and negative E-DII score groups, and it also was associated with the E-DII score. TNF-α is the most highly secreted cytokine during obesity and was the first inflammatory cytokine linking obesity-induced inflammation to IR [41,42]. This suggests that TNF-α is a more prominent biomarker of obesity-induced inflammation than 1L-8 and MCP-1, in particular for this population, with high levels of obesity. However, the non-significant association between TNF-α and markers of T2D risk suggests that TNF-α (and other inflammatory cytokines) may not be directly associated with markers of T2D risk in this population or their effects might be diminished in the presence of more detrimental risk factors, such as obesity. Accordingly, it was reported that, despite a higher inflammatory profile at the genetic, adipose and circulating level, inflammation may not be the main driver of the higher T2D risk in black populations [43]. However, inflammation acts via paracrine and autocrine effects, limiting our ability to measure their specific effects. Nonetheless, results from our study and others suggest that other risk factors, such as body fat and its distribution, SES and lifestyle factors, including physical activity, as well as their interactions may be relatively more important risk factors for T2D in this population.

In our study the E-DII score ranged from −2.51 to +1.92, which is narrower than that reported in a previous study of T2D (−5.49 to + 4.12) [21]. The differences in the E-DII range between our study and the previous study may be explained by numerous factors, including differences in sample size, age, gender representation, type and number of food-parameters used in the calculation of the DII score and quantity of the food-parameters consumed. We also propose that the narrow range in the E-DII score might be explained by the homogeneity of the sample, in terms of SES and socio-cultural background (i.e., eating similar foods and calorically similar amounts), thus resulting in a low variety of the diet, especially when taking into account energy intake. Furthermore, it is also important to note that SES factors were not significantly different between the positive and negative E-DII score groups, implying that other external factors (e.g., socio-cultural factors) might be contributing to the pro- and anti-inflammatory profile of the diet. Furthermore, those individuals consuming a pro-inflammatory diet are at high risk of developing T2D, in particular those with central obesity.

This is the first study to investigate diet-associated inflammation in relation to T2D risk, in a high-risk and understudied African population, whose lifestyle differs from other populations. Importantly, we used DXA to provide a more accurate and objective measure of whole-body composition, including estimates of VAT. Despite its strengths, our study has potential limitations. This is a cross-sectional study, thus our results do not imply causality. Also, only three out of the eight possible inflammatory cytokines were detected in all the samples and used in the analysis, thus reducing the chance of finding significant results. Furthermore, the dietary intake was measured at a single time point, thus might not reflect the long-term or seasonal changes in dietary intake and lifestyle activities of the participants. In an attempt to control and reduce the confounding effects of total energy intake between the participants, the DII was adjusted per 1000 calories. Another potential weakness of the study is the use of only 21 food parameters out of 45 for the analysis of the DII score. However, the majority of earlier studies have not used all 45 food parameters [13,20,21]. For example, both Vahid et al. [20] and Denova-Gutierrez et al. [21] who have shown positive associations between DII and prediabetes or T2D risk used 27 food items out of the possible 45 food parameters. Nonetheless, positive associations between DII and inflammation have been reported even with fewer food parameters [13,20,21], supporting the robustness of the DII in investigating the association between diet-induced inflammation and inflammatory-related diseases. Furthermore, E-DII takes into account the entire diet of the individual and measures its inflammatory potential [14], making it a better tool compared to other dietary tools that only consider dietary patterns/food-groups and single-nutrients. Nonetheless, the DII score was calibrated against a global dietary database that did not include an African population [14]; which may not capture the true inflammatory profile of an African diet. Future refinements of the DII might include expansion of the global comparative database, which may help to capture even greater dietary diversity between populations.

## 5. Conclusions

In conclusion, our findings suggest that a pro-inflammatory diet may exacerbate the effects of obesity, in particular central obesity, and that a pro-inflammatory diet may increase the risk of developing T2D. Therefore, the promotion of healthy eating might prevent or reduce central obesity and obesity-induced inflammation, and subsequently lower the risk of developing T2D in black middle-aged black SA women.

## Figures and Tables

**Table 1:** Body composition and clinical characteristics of the participants according to E-DII scores.

	Overall	Negative E-DII Group (*n* = 107)	Positive E-DII Group (*n* = 83)	*p* Value
E-DII score (minimum-maximum value)	−2.51–1.92	−2.51–0.01	0.01–1.92	
E-DII	−0.2 ± 1.0	−0.9 ± 0.6	0.6 ± 0.5	
Age (years)	53 (48–59)	54 (49–59)	54 (49–60)	0.87
Anthropometry				
Height (m)	1.6 ± 0.1	1.6 ± 0.1	1.6 ± 0.1	0.93
Weight (kg)	84.7 (73.6–96.8)	84.0 (70.4–94.2)	87.2 (76.4–100.2)	0.15
BMI (kg/m^2^)	33.5 (29.8–38.8)	33.5 (29.0–37.2)	34.2 (30.2–40.1)	0.23
BMI categories, *n* (%)				
Normal weight	18 (9.5)	10 (9.4)	8 (9.6)	0.46
Overweight	30 (15.8)	20 (18.7)	10 (12.1)	
Obese	142 (74.7)	77 (72.0)	65 (78.3)	
Waist circumference (cm)	100.1 ± 13.0	98.3 ± 12.3	102.4 ± 13.7	0.03
Waist circumference-abdominal obesity, *n* (%)				
Waist circumference (≥80 cm)	177 (93.2)	100 (93.5)	77 (92.8)	0.85
Waist circumference (<80 cm)	13 (6.8)	7 (6.5)	6 (7.2)	
Hip circumference (cm)	121.8 ± 14.5	121.0 ± 14.3	122.9± 14.7	0.21
WHR	0.8 ± 0.1	0.8 ± 0.1	0.8 ± 0.1	0.04
Body composition and body fat distribution				
Body Fat mass (kg)	39.6 (31.5 –48.2)	38.7 (29.8–46.6)	40.9 (33.1–50.8)	0.12
Body fat (%)	50.7 (46.2–54.0)	50.1 (44.8–53.8)	51.5 (47.0–54.6)	0.09
VAT area (cm^2^)	165 ± 62	155 ± 59	178 ± 64	0.01
SAT area (cm^2^)	507 ± 157	495 ± 154	521 ± 161	0.27
Clinical biomarkers				
Fasting glucose (mmol/L)	4.9 (4.3–5.5)	4.8 (4.3–5.4)	5.1 (4.3–5.7)	0.07
Fasting insulin (mUI/mL)	10.4 (5.7–18.0)	9.8 (5.7–15.7)	11.7 (5.9–18.9)	0.27
Two-hour glucose (mmol/L)	6.7 (5.3–8.1)	6.3 (5.3–7.8)	7.2 (6.0–8.6)	0.02
HbA1c (%)	5.5 (5.1–5.7)	5.4 (5.1–5.7)	5.5 (5.2–5.8)	0.48
HOMA2-IR	1.5 (0.8–2.6)	1.4 (0.8–2.2)	1.7 (0.8–2.7)	0.23
Matsuda Index (OGTT-derived insulin sensitivity)	3.6 (2.1–6.0)	3.9 (2.4–6.1)	2.9 (1.9–5.6)	0.10
TNF-α (pg/mL)	11.0 (8.0–15.1)	9.9 (7.8–14.2)	12.1 (8.7–16.7)	0.03
IL-8 (pg/mL)	5.4 (2.4–11.4)	4.8 (2.6–8.9)	6.3 (2.2–12.6)	0.16
MCP-1 (pg/mL)	436.2(325.2–627.5)	449.9 (321.2–664.7)	422.6 (330.1–595.7)	0.71

Data presented as means ± SD or median (25th–75th percentiles). BMI, body mass index; DII, dietary inflammatory index; HbA1c, glycated hemoglobin; HOMA2-IR, Homeostasis Model Assessment- insulin resistance; IL-8; Interleukin-8, MCP-1, monocyte chemotactic protein 1; OGTT, oral glucose tolerance test. SAT, subcutaneous adipose tissue; TNF-α, tumor necrosis factor alpha; VAT, visceral adipose tissue; WC, waist circumference; WHR, waist-to-hip ratio.

**Table 2:** Direct and indirect effects of E-DII on markers of T2D risk with body composition as mediators in black South African (SA) women.

Variables	Total Effect (c)		Direct Effect (c’)		Indirect Effect (αβ)		Proportion of Mediation
Mediators and Outcomes	Estimate (95%CI)	*p*-Value	Estimate (95%CI)	*p*-value	95%CI	*p*-value	%
Fasting glucose (mmol/L)	0.17 (0.01–0.34)	0.04					
via waist circumference (cm)			0.12 (−0.07–0.32)	0.21	0.04 (−0.03–0.12)	0.25	23.5
via BMI (kg/m^2^)			0.13 (−0.05–0.32)	0.16	0.04 (−0.03–0.10)	0.28	23.5
via VAT (cm^2^)			0.07 (−0.10–0.24)	0.42	0.10 (0.00–0.20)	0.04	58.8
via body fat mass (kg)			0.14 (−0.05–0.33)	0.14	0.03 (−0.03–0.08)	0.371	17.6
Fasting insulin (mUI/mL)	1.70 (0.45–2.96)	0.008					
via waist circumference (cm)			0.38 (−0.87–1.63)	0.55	1.32 (0.40–2.25)	0.005	77.6
via BMI (kg/m^2^)			0.71 (−0.51–1.93)	0.26	0.99 (0.20–1.78)	0.01	58.2
via VAT (cm^2^)			0.08 (−1.19–1.35)	0.90	1.62 (0.60–2.64)	0.002	95.3
via body fat mass (kg)			0.89 (−0.30–2.09)	0.14	0.81 (0.11–1.51)	0.02	47.6
HbA1c (%)	0.10 (0.01–0.18)	0.02					
via waist circumference (cm)			0.07 (−0.02–0.15)	0.14	0.03 (−0.00–0.06)	0.07	30.0
via BMI (kg/m^2^)			0.08 (−0.01–0.16)	0.08	0.02 (−0.01–0.05)	0.13	20.0
via VAT (cm^2^)			0.05 (−0.02–0.14)	0.18	0.04 (0.00–0.08)	0.04	40.0
via body fat mass (kg)			0.08 (−0.00–0.17)	0.06	0.01 (−0.01–0.04)	0.19	10.0
HOMA2-IR	0.25 (0.07–0.44)	0.008					
via waist circumference (cm)			0.05 (−0.15–0.26)	0.59	0.20 (0.05–0.35)	0.009	80.0
via BMI (kg/m^2^)			0.10 (−0.09–0.30)	0.30	0.15 (0.02–0.28)	0.02	60.0
via VAT (cm^2^)			0.00 (−0.20–0.21)	0.97	0.25 (0.08–0.41)	0.003	100.0
via body fat mass (kg)			0.13 (−0.05–0.32)	0.16	0.12 (0.01–0.23)	0.03	48.0
Two-hour glucose (mmol/L)	0.48 (0.10–0.86)	0.01					
via waist circumference (cm)			0.34 (−0.08–0.76)	0.11	0.14 (−0.01–0.028)	0.06	29.2
via BMI (kg/m^2^)			0.39 (−0.02–0.81)	0.06	0.09 (−0.04–0.21)	0.17	18.8
via VAT (cm^2^)			0.21 (−0.15–0.56)	0.26	0.27 (0.08–0.47)	0.006	56.3
via body fat mass (kg)			0.42 (0.00–0.83)	0.05	0.06 (−0.04–0.17)	0.27	12.5
Matsuda Index *	0.05 (0.02–0.09)	0.003					
via waist circumference (cm)			0.01 (−0.04–0.06)	0.62	0.04 (0.00–0.08)	0.03	80.0
via BMI (kg/m^2^)			0.02 (−0.02–0.07)	0.31	0.03 (−0.00–0.06)	0.05	60.0
via VAT (cm^2^)			−0.01 (−0.06–0.05)	0.85	0.05 (0.01–0.10)	0.01	100.0
via body fat mass (kg)			0.03 (−0.01–0.07)	0.16	0.02 (−0.00–0.05)	0.08	40.0

Regression coefficients c, α, β and c’ are shown in Figure 1a,b. All estimates were adjusted for the potential effects of age. BMI, body mass index; DII, dietary inflammatory index; HbA1c, glycated hemoglobin; HOMA2-IR, Homeostasis Model Assessment- insulin resistance; VAT, visceral adipose tissue. * Matsuda index was modelled as an inverse variable in order to calculate the proportion of mediation effect, thus the regression coefficients are positive instead of negative.

**Table 3:** Total, direct and indirect effects of E-DII on markers of T2D risk with inflammatory cytokines as mediators in black SA women.

Variables	Total Effect (c)		Direct Effect (c’)		Indirect Effect (αβ)	
Mediators and Outcomes	Estimate (95%CI)	*p*-Value	Estimate (95%CI)	*p*-Value	Estimate (95%CI)	*p*-Value
Fasting glucose (mmol/L)	0.17 (0.01–0.34)	0.04				
via TNF-α (pg/mL)			0.16 (−0.01–0.33)	0.07	0.01 (−0.01–0.04)	0.28
via IL-8 (pg/mL)			0.17 (0.01–0.34)	0.04	−0.00 (−0.01–0.01)	0.71
via MCP-1 (pg/mL)			0.16 (0.01–0.32)	0.04	0.01 (−0.01–0.03)	0.43
Fasting insulin (mUI/mL)	1.70 (0.45–2.96)	0.008				
via TNF-α (pg/mL)			1.55 (0.32–2.78)	0.01	0.15 (−0.06–0.36)	0.17
via IL-8 (pg/mL)			1.72 (0.47–2.97)	0.007	−0.02 (−0.14–0.11)	0.77
via MCP-1 (pg/mL)			1.66 (0.43–2.88)	0.008	0.04 (−0.13–0.21)	0.63
HbA1c (%)	0.10 (0.01–0.18)	0.02				
via TNF-α (pg/mL)			0.11 (0.02–0.19)	0.01	−0.01 (−0.02–0.00)	0.15
via IL-8 (pg/mL)			0.10 (0.02–0.18)	0.02	−0.00 (−0.01–0.00)	0.49
via MCP-1 (pg/mL)			0.10 (0.02–0.18)	0.02	−0.00 (−0.01–0.01)	0.92
HOMA2-IR	0.25 (0.07–0.44)	0.008				
via TNF-α (pg/mL)			0.23 (0.05–0.41)	0.02	0.02 (−0.01–0.06)	0.15
via IL-8 (pg/mL)			0.26 (0.07–0.44)	0.007	−0.00 (−0.02–0.02)	0.75
via MCP-1 (pg/mL)			0.25 (0.07–0.43)	0.007	0.01 (−0.02–0.03)	0.59
Two-hour glucose (mmol/L)	0.48 (0.10–0.86)	0.01				
via TNF-α (pg/mL)			0.46 (0.07–0.85)	0.02	0.02 (−0.02–0.07)	0.31
via IL-8 (pg/mL)			0.49 (0.11–0.87)	0.01	−0.01 (−0.04–0.01)	0.42
via MCP-1 (pg/mL)			0.46 (0.08–0.83)	0.02	0.03 (−0.04–0.09)	0.41
Matsuda Index *	0.05 (0.02–0.09)	0.003				
via TNF-α (pg/mL)			0.05 (0.01–0.08)	0.007	0.00 (−0.00–0.01)	0.23
via IL-8 (pg/mL)			0.05 (0.02–0.09)	0.003	−0.00 (−0.00–0.00)	0.61
via MCP-1 (pg/mL)			0.05 (0.02–0.09)	0.003	0.00 (−0.00–0.01)	0.67

Regression coefficients α, β and c’ are shown in Figure 2a,b. All estimates were adjusted for the potential effects of age. DII, dietary inflammatory index; HbA1c, glycated hemoglobin; HOMA2-IR, Homeostasis Model Assessment- insulin resistance; IL-8; Interleukin-8, MCP-1, monocyte chemotactic protein 1; TNF-α, tumor necrosis factor alpha. * Matsuda index was modelled as an inverse variable in order to calculate the proportion of mediation effect, thus the regression coefficients are positive instead of negative.

**Table 4:** Regression coefficients (95% CIs) for the association between E-DII and measures of body composition and inflammatory cytokines in black SA women.

Mediator Variables	Estimate	95%CI	*p*-Value
Waist circumference (cm)	3.27	1.39–5.16	0.001
BMI (kg/m^2^)	1.54	0.52–2.55	0.003
VAT area (cm^2^)	16.88	8.30–25.46	<0.0001
Body fat mass (kg)	2.49	0.65–4.32	0.008
TNF-α (pg/mL)	0.98	0.13–1.82	0.02
IL-8 (pg/mL)	0.98	−1.09–3.01	0.36
MCP-1 (pg/mL)	25.73	−17.47–68.92	0.24

Estimates for the association between E-DII and body composition and inflammatory cytokines represent the regression coefficients for Path α Figure 2b. All estimates were adjusted for the potential effects of age. BMI, body mass index; DII, dietary inflammatory index; IL-8; Interleukin-8, MCP-1, monocyte chemotactic protein 1; TNF-α, tumor necrosis factor alpha; VAT, visceral adipose tissue.

**Table 5:** Regression coefficients (95% CIs) for the associations between body composition and inflammatory cytokines and markers of T2D risk in black SA women.

	**Fasting Glucose**			**Fasting Insulin**			**HbA1c**		
	**Estimate**	**95%CI**	***p*-Value**	**Estimate**	**95%CI**	***p*-Value**	**Estimate**	**95%CI**	***p*-Value**
Waist circumference (cm)	0.01	−0.01–0.03	0.22	0.40	0.23–0.57	<0.0001	0.01	0.00–0.02	0.04
BMI (kg/m^2^)	0.02	−0.02–0.06	0.25	0.65	0.34–0.95	<0.0001	0.01	−0.00–0.03	0.09
VAT area (cm^2^)	0.01	0.00–0.01	0.02	0.10	0.06–0.13	<0.0001	0.00	0.00–0.00	0.02
Body fat mass (kg)	0.01	−0.01–0.03	0.33	0.33	0.17–0.48	<0.0001	0.01	−0.00–0.01	0.14
TNF-α (pg/mL)	0.01	−0.01–0.03	0.20	0.15	−0.04–0.34	0.11	−0.01	−0.02–0.00	0.06
IL-8 (pg/mL)	−0.00	−0.01–0.01	0.63	−0.02	−0.13–0.09	0.73	−0.00	−0.01–0.00	0.14
MCP-1 (pg/mL)	0.00	−0.00–0.00	0.29	0.00	−0.00–0.01	0.56	−0.00	−0.00–0.00	0.92
	**HOMA2-IR**			**Two-Hour Glucose**			Matsuda Index *		
	**Estimate**	**95%CI**	***p*-Value**	**Estimate**	**95%CI**	***p*-Value**	**Estimate**	**95%CI**	***p*-Value**
Waist circumference (cm)	0.06	0.03–0.09	<0.0001	0.04	0.01–0.08	0.03	0.01	0.00–0.02	0.008
BMI (kg/m^2^)	0.10	0.04–0.15	<0.0001	0.06	−0.01–0.12	0.10	0.02	0.00–0.03	0.02
VAT area (cm^2^)	0.01	0.01–0.02	<0.0001	0.02	0.01–0.02	<0.0001	0.00	0.00–0.00	0.001
Body fat mass (kg)	0.05	0.02–0.08	<0.0001	0.03	−0.01–0.06	0.20	0.01	0.00–0.01	0.03
TNF-α (pg/mL)	0.02	−0.00–0.05	0.10	0.03	−0.02–0.07	0.26	0.00	−0.00–0.01	0.13
IL-8 (pg/mL)	−0.00	−0.02–0.01	0.70	−0.01	−0.03–0.01	0.19	−0.00	−0.00–0.00	0.39
MCP-1 (pg/mL)	0.00	−0.00–0.00	0.49	0.00	−0.00–0.00	0.16	0.00	−0.00–0.00	0.58

Regression coefficients correspond to Path β in Figure 2b. All estimates were adjusted for the potential effects of age. BMI, body mass index; DII, dietary inflammatory index; HbA1c, glycated hemoglobin; HOMA2-IR, Homeostasis Model Assessment- insulin resistance; IL-8; Interleukin-8, MCP-1, monocyte chemotactic protein 1; TNF-α, tumor necrosis factor alpha; VAT, visceral adipose tissue. * Matsuda index was modelled as an inverse variable in order to calculate the proportion of mediation effect, thus the regression coefficients are positive instead of negative.

## Supplementary Materials

The following are available online at https://www.mdpi.com/2072-6643/11/6/1246/s1, Table S1: Demographic, behavioral and lifestyle factors characteristics of the participants, Table S2: Nutrient intake between the positive and negative E-DII score groups.
