# Supplementary material for: Adiposity Mediates the Association between the Dietary Inflammatory Index and Markers of Type 2 Diabetes Risk in Middle-Aged Black South African Women
Source: Nutrients. 2019 May 31;11(6):1246. doi: 10.3390/nu11061246 (PMC6628082; doi:10.3390/nu11061246)
Supplement: Supplementary file 1 [file nutrients-11-01246-s001.pdf]

Supplementary information

Table S1. Demographic, behavioral and lifestyle factors characteristics of the participants.

	Overall	Negative E-DII group (n=107)	Positive E-DII group (n=83)	p-value
**Sociodemographic factors**				
**Housing density (persons/room)**	1 (0.7-1.3)	0.9 (0.6-1.2)	1.0 (0.8-1.3)	0.12
**Asset index (out of 12 items)**	9 (7-11)	9 (7-11)	9 (7-11)	0.87
**Household Food insecurity score**	4 (0-9)	3 (0-8)	4 (0-10)	0.21
**Marital status**				
Married	104 (54.7)	56 (52.3)	48 (57.8)	0.45
Unmarried	86 (45.3)	51 (47.7)	35 (42.2)	
**Highest level of education, n (%)**				
Primary education	29 (15.3)	16 (15.0)	13 (15.7)	0.22
Some secondary education	89 (46.8)	44 (41.1)	45 (54.2)	
Secondary education completed (grade 12)	41 (21.6)	28 (26.2)	13 (15.7)	
Tertiary education	31 (16.3)	19 (17.8)	12 (14.5)	
**Employment, n (%)**				
Employment	100 (52.6)	58 (54.2)	42 (50.6)	0.622
Unemployed	90 (47.4)	49 (45.8)	41 (49.4)	
**Monthly income, n (%)**				
1=No income	1 (0.5)		1 (1.2)	0.51
2=R1-4999	101 (54.0)	59 (56.2)	42 (51.2)	
3=R5000-9999	39 (20.9)	20 (19.0)	19 (23.2)	
4=R10000-19999	34 (18.2)	21 (20.0)	13 (15.9)	

5= (>R20000)	12 (6.4)	5 (4.8)	7 (8.5)	
**3 participants did not disclose their monthly income*				
**Behavioral and lifestyle factors**				
**Smoking status, n (%)**				
Never smoked	204 (96.3)	104 (97.2)	79 (95.2)	0.46
Current smoker	8 (3.7)	3 (2.8)	4 (4.8)	
**Alcohol intake, n (%)**				
Yes	49 (25.8)	24 (22.4)	25 (30.1)	0.23
No/Never	141 (74.2)	83 (77.6)	58 (69.9)	
**Physical activity data**				
Light (hr/d)	6.4 (5.4-7.4)	6.6 (5.5-5.2)	6.1 (5.2-7,2)	0.14
Moderate (mins/d)	37.4 (21.4-51.9)	41.5 (21.5-50.8)	33.7 (18.7-52.8)	0.31
Vigorous (mins/d)	0.1 (0.0-0.5)	0.2 (0.0-0.6)	0.0 (0.0-0.3)	0.19
Total MVPA (mins/d)	37.9 (21.6-53.2)	43.8 (21.7-53..0)	33.7 (18.7-53.5)	0.23
MVPA (% per day)	4.3 (2.3-5.9)	4.9 (2.4-6.0)	3.7 (2.2-5.7)	0.25
Steps per day	6590.4 (4871.7-8583.7)	6945.7 (5063.9-8736.1)	6203.0 (4792.8-8249.0)	0.12
**Sedentary behavior**				
Sedentary (hr/d)	8.5 ± 2.2	8.5 ± 2.3	8.4 ± 2.0	0.69
Sedentary (% per day)	50.8 ± 12.9	50.8 ± 13.7	50.9 ± 11.8	0.99
Standing (% per day)	36.9 ± 10.6	36.6 ± 11.4	37.2 ± 9.4	0.70
Stepping (% per day)	12.3 ± 4.2	12.6 ± 4.3	11.9 ± 4.0	0.31

Data presented as means ± SD, median (25th-75th percentiles) or frequency (%). E-DII, energy-adjusted dietary inflammatory index; MVPA, moderate-vigorous physical activity.

Table S2. Nutrient intake between the positive and negative E-DII score groups.

		Negative E-DII group (n=107)	Positive E-DII group (n=83)	p-value
Number (%)	**Overall**	**107 (56.3%)**	**83(43.7%)**	
Energy (kcal/d)	1464.8 (1115.0-1959.1)	1461.9 (1115.0-1962.1)	1490.3 (1111.1-1905.9)	0.66
Protein (g/d)	36.6 (28.7-52.5)	38.8 (29.4-57.7)	34.9 (26.8-46.8)	0.03
Protein (%E)	10.2 (8.5-12.6)	11.1 (9.4-13.0)	9.2 (7.8-10.8)	<0.0001
Total fat (g/d)	60.5 (45.4-85.9)	58.2 (41.5-79.6)	71.8 (52.5-99.1)	0.004
Total fat (%E)	38.1 (32.2-43.9)	35.3 (29.9-39.9)	42.4 (36.5-48.4)	<0.0001
Saturated fat (g/d)	14.2 (11.0-20.8)	13.2 (9.9-19.2)	17.1 (11.7-22.5)	0.007
Saturated fat (%E)	8.9 (7.6-10.4)	8.2 (7.3-9.5)	10.0 (8.6-11.3)	<0.0001
Monounsaturated fatty acid (g/d)	20.4 (14.7-29.8)	19.6 (13.6-27.2)	21.8 (15.7-33.9)	0.03
Monounsaturated fatty acid (%E)	12.6 (10.3-14.9)	11.9 (9.9-13.9)	13.6 (11.3-15.8)	0.001
Polyunsaturated fatty acid (g/d)	22.6 (14.7-30.7)	18.8 (12.0-28.4)	24.4 (17.7-34.2)	0.002
Polyunsaturated fatty acid (%E)	12.6 (8.8-16.9)	11.0 (8.4-14.4)	15.8 (10.3-19.0)	0.0001
Carbohydrates (g/d)	181.4 (135.6-247.3)	194.0 (142.5-256.9)	168.6 (125.6-239.4)	0.18
Carbohydrates (%E)	50.8 (45.5-56.7)	52.3 (48.1-58.3)	47.0 (41.4-54.0)	<0.0001
Alcohol	0.0 (0.0-0.0)	0.0 (0.0-0.0)	0.0 (0.0-0.0)	0.14
Cholesterol (mg)	132.3 (83.8-217.0)	132.6 (85.7-208.1)	132.1 (80.5-240.9)	0.89
Fiber (g/d)	12.0 (8.4-17.3)	14.5 (10.4-20.1)	9.2 (7.0-13.1)	<0.0001
Iron (mg/d)	8.5 (6.3-12.1)	9.7 (7.0-13.7)	7.8 (5.7-10.2)	0.005
Magnesium (mg/d)	159.4 (118.2-210.0)	175.7 (129.2-250.8)	136.3 (105.2-200.4)	0.002
Zinc (mg/d)	6.5 (5.0-9.0)	7.43 (5.7-10.3)	6.0 (4.3-7.5)	<0.0001)
Vitamin A (Retinol equivalent) (µg/d)	617.9 (354.7-1007.8)	664.0 (393.8-1039.9)	590.5 (300.4-1007.8)	0.21
Thiamine (mg/d)	1.0 (0.8-1.5)	1.1 (0.8-1.6)	1.0 (0.7-1.3)	**0.03**
Ribloflavin (mg/d)	0.8 (0.6-1.1)	0.7 (0.6-1.2)	0.8 (0.5-1.1)	0.57
Niacin (mg/d)	13.0 (9.8-18.2)	15.0 (10.9-21.0)	10.8 (8.5-15.5)	<0.0001
Vitamin B6 (mg/d)	1.8 (1.3-2.8)	2.0 (1.5-3.4)	1.4 (1.0-2.2)	<0.0001
Vitamin B12	1.9 (1.2-3.5)	2.0 (1.2-3.3)	1.9 (1.2-3.7)	0.78
Vitamin C (mg/d)	29.9 (15.7-65.3)	38.6 (17.2-69.6)	23.5 (12.9-53.0)	0.009
Vitamin D (µg/d)	3.9 (2.5-5.4)	3.8 (2.2-5.3)	4.1 (3.0-5.6)	0.22
Vitamin E (mg/d)	13.4 (8.0-20.5)	11.8 (6.9-18.5)	14.7 (10.8-24.3)	0.008

Values expressed as median (25th-75th percentiles).
